# Manganese Exposure and Neurocognitive Outcomes in Rural School-Age Children: The Communities Actively Researching Exposure Study (Ohio, USA)

**DOI:** 10.1289/ehp.1408993

**Published:** 2015-04-22

**Authors:** Erin N. Haynes, Heidi Sucharew, Pierce Kuhnell, Jody Alden, Mary Barnas, Robert O. Wright, Patrick J. Parsons, Kenneth M. Aldous, Meredith L. Praamsma, Caroline Beidler, Kim N. Dietrich

**Affiliations:** 1 Department of Environmental Health, College of Medicine, University of Cincinnati, Cincinnati, Ohio, USA; 2 Division of Biostatistics and Epidemiology, Cincinnati Children’s Hospital Medical Center, Cincinnati, Ohio, USA; 3 Department of Psychology, Marietta College, Marietta, Ohio, USA; 4 Icahn School of Medicine at Mount Sinai, New York, New York, USA; 5 Wadsworth Center, New York State Department of Health, Albany, New York, USA; 6 Department of Environmental Health Sciences, University at Albany, School of Public Health, Albany, New York, USA; 7 Neighbors for Clean Air, Marietta, Ohio, USA

## Abstract

**Background:**

Manganese (Mn) plays a vital role in brain growth and development, yet excessive exposure can result in neurotoxicity. Marietta, Ohio, is home to the nation’s longest-operating ferromanganese refinery, and community concern about exposure led to the development of the research study.

**Objectives:**

Our overall goal was to address the community’s primary research question: “Does Mn affect cognitive development of children?” We evaluated the relationships between Mn exposure as measured by blood and hair Mn, along with other neurotoxicants including blood lead (Pb) and serum cotinine, and child cognition.

**Methods:**

Children 7–9 years of age were enrolled (*n* = 404) in the Communities Actively Researching Exposure Study (CARES) from Marietta and Cambridge, Ohio, and their surrounding communities from October 2008 through March 2013. Blood and hair were analyzed for Mn and Pb, and serum was analyzed for cotinine. We used penalized splines to assess potential nonlinear associations between biological measures and IQ subscale scores, followed by multivariable regression models with categorical variables based on quartiles of the distribution for biological measures with nonlinear associations and continuous variables for biological measures with linear associations.

**Results:**

Geometric mean blood (*n* = 327) and hair Mn (*n* = 370) concentrations were 9.67 ± 1.27 μg/L and 416.51 ± 2.44 ng/g, respectively. After adjusting for potential confounders, both low and high blood and hair Mn concentrations were associated with lower Full Scale IQ and subscale scores, with significant negative associations between the highest quartile and middle two quartiles of blood Mn (β –3.51; 95% CI: –6.64, –0.38) and hair Mn (β –3.66; 95% CI: –6.9, –0.43%) and Full Scale IQ.

**Conclusions:**

Both low and high Mn concentrations in blood and hair were negatively associated with child IQ scores. Serum cotinine was negatively associated with child cognitive function.

**Citation:**

Haynes EN, Sucharew H, Kuhnell P, Alden J, Barnas M, Wright RO, Parsons PJ, Aldous KM, Praamsma ML, Beidler C, Dietrich KN. 2015. Manganese exposure and neurocognitive outcomes in rural school-age children: the Communities Actively Researching Exposure Study (Ohio, USA). Environ Health Perspect 123:1066–1071; http://dx.doi.org/10.1289/ehp.1408993

## Introduction

Manganese (Mn) is an essential trace element that plays a vital role in normal growth and development, particularly brain development (Aschner 2000; Erikson and Aschner 2006). Although ingested Mn is under tight homeostatic control (Papavasiliou et al. 1966), inhaled Mn can bypass the biliary excretion mechanism and enter the brain through facilitated diffusion and active transport across the blood–brain barrier (Aschner 2006; Crossgrove et al. 2003; Davis 1999; Elder et al. 2006; Rabin et al. 1993) or passive transport from the olfactory bulb to the cerebral cortex (Dorman et al. 2002; Elder et al. 2006). The brain may be particularly sensitive to Mn overexposure; blood and hair concentrations of Mn have been associated with deficits in neuropsychological and motor functions (Hernández-Bonilla et al. 2011; Long et al. 2014; Lucchini et al. 2012; Mergler et al. 1999; Meyer-Baron et al. 2013; Standridge et al. 2008; Torres-Agustín et al. 2013; Zoni and Lucchini 2013). Recently, an inverse U-shaped association was reported between blood Mn concentrations and the Mental Development Index within the Bayley Scales of Infant Development, suggesting possible adverse effects of both low and high exposures on neurodevelopment (Claus Henn et al. 2010).

Marietta, Ohio, is home to the longest operating ferromanganese refinery in North America, Eramet Marietta, Inc. (EMI; (http://www.erametmarietta.com/). EMI, a French-owned company, refines Mn ore into either refined ferromanganese or silicomanganese, which are used primarily in steel production. EMI has reported emitting hundreds of thousands of pounds of Mn per year into the air (U.S. EPA 2011). We have developed a community-based participatory research (CBPR) partnership with residents from Marietta and its surrounding communities to conduct an epidemiologic investigation to address their leading environmental public health concern: “Does Mn affect cognitive development of children” (Haynes et al. 2011)?

Given that exposures do not occur in isolation, we included other well-documented neurotoxicants in our analyses, including lead (Pb) and environmental tobacco smoke (ETS). The neurodevelopmental consequences of Pb exposure in children are well documented (Chen et al. 2007; Dietrich 2010; Lanphear et al. 2000). Childhood blood Pb concentrations < 10 μg/dL have been associated with impairments to cognitive function (Canfield et al. 2003; Jusko et al. 2008; Lanphear et al. 2005; Schwartz 1994; Téllez-Rojo et al. 2006). Although federal bans on Pb in paint and gasoline have resulted in dramatic reductions in childhood blood Pb concentrations, no threshold for the effects of Pb exposure on children’s cognition and behavior has been identified (Bellinger 2008). ETS exposure during childhood has been associated with deficits in cognition and intelligence (Eskenazi and Bergmann 1995; Yolton et al. 2005), and Appalachian regions, where this study population is located, within the United States have higher tobacco use prevalence than non-Appalachian regions (Wewers et al. 2000). In this study we assessed the impact of Mn exposure on neurocognition in a cohort of school-age children residing in rural eastern Ohio Appalachian communities, along with exposure to Pb and ETS and key potential confounders that may be related to the neurodevelopmental outcomes under study.

## Methods

*Study participants*. Children were recruited to participate in the Communities Actively Researching Exposure Study (CARES) if they were 7, 8, or 9 years of age and resided in Marietta or Cambridge, Ohio, and their surrounding communities throughout their life with no plans to move for at least 1 year (Rugless et al. 2014). In addition, their biological mother must have resided in the catchment area during her pregnancy with the index child. Families were recruited for participation in CARES using a volunteer sampling strategy from October 2008 through March 2013. Recruitment postcards were sent home with children through schools, and advertisements were aired on local radio and printed in local newspapers. Children were excluded if they did not meet the eligibility criteria or had a health condition that impeded their ability to participate in the behavioral assessment testing, such as a significant visual, auditory, or motor impairment. The University of Cincinnati Institutional Review Board approved this study. All parents and children signed an informed consent, and the children also signed an informed assent.

*Specimen collection and analysis*. All biological specimens and neurocognitive assessments were collected during the same study visit.

Approximately 20 strands of hair were collected from the occipital region, cut with ceramic scissors as close to the scalp as possible. It was ensured that the hair was at least 1 cm in length for analysis. Long hair was trimmed to 6 cm and taped toward the non-scalp-side end of the hair shaft onto an index card with an arrow pointing in the direction of the scalp end on the index card. The card with the taped hair sample was placed into a pre-labeled envelope and stored at room temperature until shipped to the Channing Trace Metals Laboratory, Brigham and Women’s Hospital, Harvard T.H. Chan School of Public Health for analysis in Boston, Massachusetts. The samples were first washed in a 1% Triton^TM^ X-100 solution and then digested using concentrated HNO_3_. Acid digestates were then analyzed by inductively coupled plasma–mass spectrometry (ICP-MS) using previously described methods (Wright et al. 2006). The method detection limit (MDL) for Mn in hair was < 2 ng/g.

Venous whole-blood specimens were collected from the antecubital vein in 3-mL purple top (K_2_EDTA) tubes certified by the analyzing laboratory for trace element analysis. Specimens were refrigerated at 5°C until they were shipped to the Laboratory of Inorganic and Nuclear Chemistry at the New York State Department of Health’s (NYS DOH) Wadsworth Center for analysis in Albany, New York.

Blood specimens were analyzed for Mn using graphite furnace atomic absorption spectrometry (GFAAS; PerkinElmer® model 4100 ZL) equipped with a transversely heated graphite atomizer and a longitudinal Zeeman background correction system (PerkinElmer® Life and Analytical Sciences, Shelton, CT) using previously described method and quality-control measures (Praamsma et al. 2011, 2012). The MDL for Mn in blood was 1.5 μg/L.

Blood Pb was determined by ICP-MS (Palmer et al. 2006) using a method optimized and validated for biomonitoring purposes as described elsewhere (Birdsall et al. 2010; McKelvey et al. 2007). A PerkinElmer® Sciex ELAN DRC Plus ICP-MS instrument equipped with a Burgener Teflon MiraMist® nebulizer (Burgener Research Inc., Mississauga, ON, Canada) and a Cinnabar spray chamber (Glass Expansion, West Melbourne, VIC, Australia) and operated in standard mode was used for all blood Pb measurements. The between-run precision for blood Mn based on internal quality control data was 7.0% relative standard deviation at 8.5 μg/L and 2.7% RSD at 23.1 μg/L. The between-run precision for blood Pb was 2.8% at 3.5 μg/dL. The MDL for blood Pb was 0.04 μg/dL.

Serum cotinine levels were also measured at the Wadsworth Center, using a high-throughput 96-well plate format sample preparation, and then analyzed using an isotope dilution, liquid chromatography/tandem mass spectrometry (LC/MS/MS) method. The method used is a modification of techniques used by the Centers for Disease Control and Prevention (CDC) for the National Health and Nutrition Examination Survey (NHANES) (Bernert et al. 1997) and New York State Wadsworth Laboratories for the NYC Health and Nutrition Examination Survey studies (Ellis et al. 2009).

Each serum specimen was equilibrated with a trideuterated cotinine internal standard solution and extracted using a 96-well Bond Elut Plexa Solid Phase Extraction (SPE) plate (Varian, Palo Alto, CA). The acetonitrile sample extract was taken to dryness, reconstituted in 96%/4% acetonitrile/water solution, and analyzed by LC/MS/MS using electrospray ionization. The instrumental systems comprised a Shimadzu Prominence LC with a Phenomonex Luna Hilic (100 × 2.00 mm) column and an AB Sciex API 4000 triple quadrupole mass spectrometer operated in electrospray ionization positive ion mode using multiple reaction monitoring (MRM) detection. Three quality control pools were used at low, medium, and high target cotinine concentrations of 0.173, 1.61, and 15.7 ng/mL. Final results were blank corrected using the mean batch blank value. The MDL for this method was 0.05 ng/mL cotinine in serum. For values below the MDL, machine readings provided by the laboratory were used in statistical analyses.

*Neurocognitive assessment*. A registered nurse from within the Marietta community conducted the neurocognitive assessments of the child (J.A.). The nurse was trained by an experienced developmental neuropsychologist and quality control was maintained throughout the investigation through periodic review of videotapes of assessment sessions (K.N.D.). The Wechsler Intelligence Scale for Children-IV (WISC-IV) was administered at the initial study visit at the time of biological sample collection. The WISC-IV is a standardized, individually administered instrument for assessing the cognitive ability of children 6–16 years of age (Wechsler 2003). The WISC-IV provides an overall score (Full Scale IQ) along with four major domains of intellectual functioning that include Perceptual Reasoning, Processing Speed, Working Memory, and Verbal Comprehension.

*Other risk factors and potential confounders*. The IQ of the primary caregiver was assessed using the Wechsler Abbreviated Scale of Intelligence (WASI) (Wechsler 1999). The Parenting Relationship Questionnaire (PRQ) (Reynolds and Kamphaus 2004) was used to assess qualities of the child’s rearing environment. The PRQ provides *t*-scores for the following domains: Attachment, Communication, Discipline Practices, Involvement, Parenting Confidence, School Satisfaction, and Relational Frustration. A separate aliquot of serum was analyzed for ferritin by the Marietta Memorial Hospital (MMH) in a CLIA (Clinical Laboratory Improvement Amendments)–certified clinical laboratory by an Abbott Architect ci8200 Integrated System Chemistry analyzer. The serum specimen was refrigerated at 5°C until transferred to MMH for analysis on the day of specimen collection. Additional demographic/socioeconomic factors included child age, child sex, child birth weight, whether or not the child spent time in the intensive care unit (ICU) after birth, whether or not the child’s home was owned or rented, and parent education using the Barratt Simplified Measure of Social Status (BSMSS) (Barratt 2006; Davis et al. 1991).

*Statistical analysis*. Univariate descriptive statistics and plots were used to examine data distributions. The following variables with skewed distributions were transformed using the natural logarithm: hair Mn, serum cotinine, and serum ferritin. Potential nonlinear associations between biological measures and WISC-IV outcomes were examined using penalized splines in generalized additive models with covariates included as linear terms. The degree of smoothing for the splines was set at 3, unless a lower degree was indicated by generalized cross validation. For untransformed blood Mn, we truncated the top 3% of blood Mn values (four values between 17.2 and 18.8) to ensure that the shape of the relationship was not influenced by outlying values.

For each WISC-IV outcome we fit a multivariable linear regression model. For the biological measures, continuous terms for blood Pb and serum cotinine were included in the model to evaluate linear associations and indicator variables for quartiles of the blood Mn, and hair Mn distributions were included to evaluate nonlinear associations. A step-wise backward elimination approach was used to obtain the most parsimonious final models. During the model reduction process, hair Mn, blood Mn, serum cotinine, blood Pb, and community residence of participants (Marietta or Cambridge and their surrounding areas) were retained in all models. Any other covariates that were either statistically significantly associated with the outcome (*p* < 0.05) or resulted in > 10% change in the estimate of either Mn exposure variables when removed were also retained. Participants with missing data for any model covariates were excluded from that model. All statistical analyses were completed using SAS version 9.4 (SAS Institute Inc., Cary, NC) and R version 2.10.1 (R Core Team 2014).

## Results

*Participant characteristics*. The vast majority of our study participants were Caucasian (non-Hispanic white; 94%; Table 1). The majority of the parents (82%) had > 12 years of education. Biological measures of blood Pb, blood Mn, and hair Mn [geometric mean (GM) (GSD)] were 0.82 (1.58 μg/dL), 9.67 (1.27μg/L), and 416.51 (2.44 ng/g), respectively (Table 1). GM of serum cotinine was 0.08 (7.84 μg/L) μg/L. Half of the serum cotinine samples were below the MDL. Urine was collected and analyzed on 170 participants, but due to the high number of these samples below the MDL (0.5 μg/L; nearly 98%), we discontinued urinary Mn analyses. We examined the correlation among the biomarkers, and those that were significantly correlated included blood Mn and serum ferritin (*r* = –0.19, *p* < 0.01), blood Mn and blood Pb (*r* = –0.13, *p* = 0.02), hair Mn and serum cotinine (*r* = 0.25, *p* < 0.0001), hair Mn and blood Pb (*r* = 0.22, *p* < 0.01), and serum cotinine and blood Pb (*r* = 0.34, *p* < 0.0001). Blood Mn and hair Mn were not significantly correlated (*r* = 0.002, *p* = 0.97).

**Table 1:** Participant characteristics from the CARES cohort.

Characteristic	Total cohort (*n = *404)
Child measures
Age (years)	8.37 ± 0.91
Child’s sex
Female	187 (46)
Male	217 (54)
Race/ethnicity
Caucasian^*a*^	379 (94)
Hispanic^*b*^	6 (1)
Other	19 (5)
Community
Marietta and surrounding area	321 (80)
Cambridge and surrounding area	81 (20)
Birth weight (g)	3,392 ± 613
ICU after birth	29 (7)
Biological measures [GM (GSD)]
Hair Mn (ng/g), *n* = 370	416.51 (2.44)
Blood Mn (μg/L), *n* = 327	9.67 (1.27)
Blood Pb (μg/dL), *n* = 327	0.82 (1.58)
Serum cotinine (μg/L), *n* = 329^*c*^	0.08 (7.84)
Serum ferritin (ng/mL) *n* = 310	34.42 (1.67)
Parent measures
Parent education
> 12 years	330 (82)
≤ 12 years	72 (18)
Home ownership
Owned	309 (77)
Rented	95 (33)
PRQ *t*-scores, *n* = 402
Attachment	51.4 ± 9.6
Communication	51.9 ± 9.3
Discipline Practices	50.0 ± 10.4
Involvement	53.9 ± 10.5
Parenting Confidence	51.8 ± 9.7
School Satisfaction	49.2 ± 12.1
Relational Frustration	49.1 ± 9.2
Values are mean ± SD or *n* (%), unless otherwise indicated. ^***a***^Non-Hispanic white. ^***b***^Hispanic ethnicity regardless of race. ^***c***^*n *= 166 (50%) serum cotinine values below MDL.	

**Figure 1:**
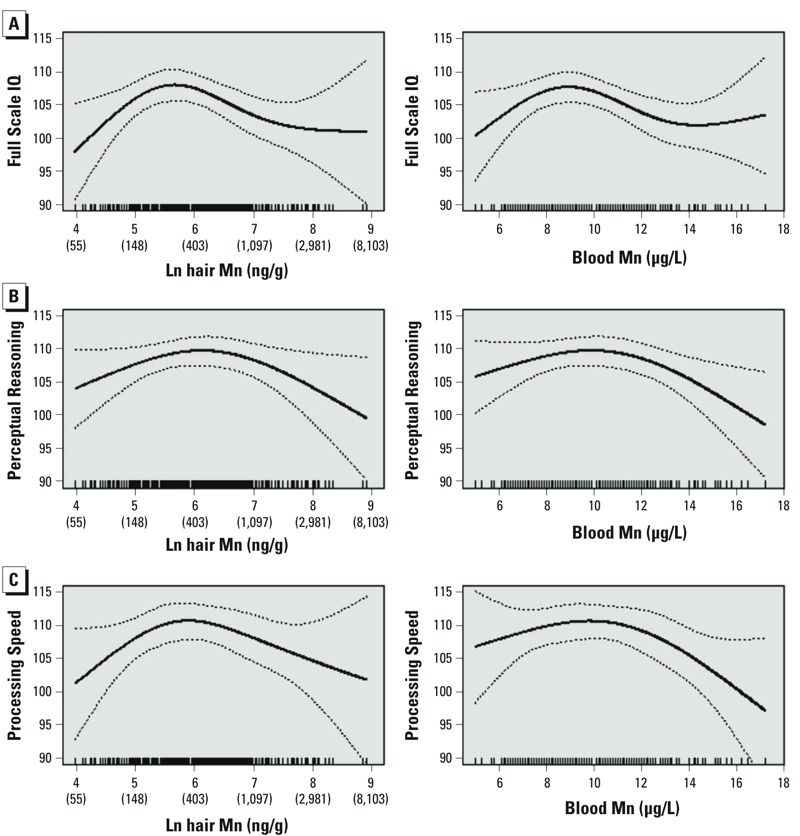
Penalized splines for hair and blood Mn levels in association with WISC-IV outcome measures. All models include both hair Mn and blood Mn, plus ln serum creatinine, blood Pb, and community residence. (*A*) Full Scale IQ (*n *= 295), also adjusted for sex, parent’s IQ, parent education, parent confidence *t*-score; (*B*) Perceptual Reasoning (*n *= 298) also adjusted for parent’s IQ; and (*C*) Processing Speed (*n *= 272), also adjusted for sex, ln serum ferritin, parent confidence *t*-score, birth weight. The solid line represents the estimate with the 95% confidence interval indicated by dotted lines. The distributions of Mn levels are indicated by vertical lines on the *x*-axis.

*Association between biomarkers and cognitive function*. The generalized linear models revealed nonlinear associations between Mn (hair and blood) and WISC-IV outcomes (Full Scale IQ: hair *p* = 0.01 and blood *p* = 0.041; Perceptual Reasoning: hair *p* = 0.09 and blood *p* = 0.04; Processing Speed: hair *p* = 0.08 and blood *p* = 0.06) (Figure 1). To account for this nonlinear association and to evaluate the upper and lower extremes of Mn levels, we fit a multivariable regression model with indicator variables for the lowest and highest quartiles of Mn (hair and blood) with the reference group set to the middle two quartiles of Mn distribution (i.e., within the 25th to 75th percentiles) (Table 2). The highest quartile of Mn, both hair and blood, was significantly associated with lower mean Full Scale IQ scores compared to the middle two quartiles of Mn. Mean Full Scale IQ was lower in the lowest quartile than the middle two quartiles of Mn, hair, and blood, but did not reach statistical significance. For Perceptual Reasoning, both the lowest and highest quartiles of hair Mn and the highest quartile of blood Mn were associated with significantly lower mean scores compared to the middle two quartiles. The highest quartile of blood Mn was also associated with lower mean Processing Speed compared with the middle two quartiles. Although associations were not statistically significant, scores for Working Memory and Verbal Comprehension also were lower, on average, among children with the lowest and highest values of hair and blood Mn, compared with children in the middle two quartiles of each exposure. In the multivariable models, a 1-unit increase in blood Pb was associated with significantly lower Processing Speed [β = –3.53; 95% confidence interval (CI): –6.95, –0.12], and ln-transformed serum cotinine was significantly associated with lower scores for Full Scale IQ and all domains except Processing Speed (e.g., β = –1.42; 95% CI: –2.23, –0.60 for Full Scale IQ).

**Table 2:** Multivariable linear regression models for WISC-IV outcomes.*^a^*

Exposure	*n*^*a*^	Full Scale IQ^*b*^ β (95% CI)	Perceptual Reasoning^*c*^ β (95% CI)	Processing Speed^*d*^ β (95% CI)	Working Memory^*e*^ β (95% CI)	Verbal Comprehension^*f*^ β (95% CI)
Hair Mn (ng/g)
< 207.2	67–75	–2.67 (–5.87, 0.53)	–4.32 (–7.82, –0.82)	0.92 (–2.87, 4.72)	–2.41 (–5.93, 1.10)	–1.13 (–4.22, 1.97)
207.2–747.0	64–69	Reference	Reference	Reference	Reference	Reference
> 747.0	141–153	–3.66 (–6.90, –0.43)	–4.51 (–8.05, –0.98)	–2.68 (–6.48, 1.11)	–1.81 (–5.35, 1.73)	–1.48 (–4.59, 1.64)
Blood Mn (μg/L)
< 8.2	62–69	–2.14 (–5.37, 1.09)	–3.32 (–6.87, 0.23)	–1.06 (–4.90, 2.77)	–1.73 (–5.28, 1.81)	–1.76 (–4.88, 1.36)
8.2–11.2	71–78	Reference	Reference	Reference	Reference	Reference
> 11.2	139–151	–3.51 (–6.64, –0.38)	–3.50 (–6.88, –0.12)	–4.17 (–7.85, –0.49)	–2.71 (–6.12, 0.70)	–1.13 (–4.13, 1.87)
Model *R*^2^		0.27	0.20	0.19	0.14	0.23
All models include both hair Mn and blood Mn, plus ln serum creatinine, blood Pb, and community. ^***a***^The *n* for each exposure model varies due to covariates. ^***b***^Also adjusted for sex, parent’s IQ, parent education, Parent Confidence *t*-score. ^***c***^Also adjusted for parent’s IQ. ^***d***^Also adjusted for sex, ln serum ferritin, Parent Confidence *t*-score, birth weight. ^***e***^Also adjusted for sex, parent’s IQ, Parent Confidence *t*-score. ^***f***^Also adjusted for parent’s IQ, Discipline Practices *t*-score, Attachment *t-*score.						

## Discussion

These findings suggest that both high and low levels of Mn may affect child neurodevelopment. Full Scale IQ scores among children in the highest quartile of blood Mn (> 11.2 μg/L) were significantly lower than scores in children with blood Mn concentrations between 8.2 and 11.2 μg/L (–3.51 points; 95% CI: –6.64, –0.38). Children within the lowest quartile of blood Mn (< 8.2 μg/L) also had lower Full Scale IQ than children in the reference group, although the association was not statistically significant (–2.14 points; 95% CI: –5.37, 1.09). The Perceptual Reasoning and Processing Speed subscales had the strongest negative associations with blood Mn. This inverted U-shaped association between blood Mn and health outcomes has been observed previously between maternal blood Mn and infant birth weight (Eum et al. 2014; Zota et al. 2009) and blood Mn and mental development (Claus Henn et al. 2010) in 270 12-month-old children.

There are currently no age-specific reference concentrations for blood Mn. The Agency for Toxic Substances and Disease Registry (ATSDR) reports normal values of 4–14 μg/L for adults (ATSDR 2000). The NHANES included blood Mn for the first time during the 2011–2012 (CDC 2014). GM blood Mn for children varied slightly by age with a decrease of 1 μg/L from ages 1–5 to ≥ 20 years: ages 1–5 (GM = 10.7; 95% CI: 10.2, 11.2); ages 6–11 (GM = 10.3; 95% CI: 9.98 10.6); ages 12–19 (GM = 10.2; 95% CI: 9.67 10.5); ages ≥ 20 (GM = 9.09; 95% CI: 8.94 9.24) (CDC 2014). Takser and colleagues found significantly higher blood Mn levels in cord blood than adult blood (Takser et al. 2004), most likely due to developmental life stage Mn requirements for normal growth and development. GM blood Mn concentration in children in this study, 9.67 μg/L, compares with the NHANES 2011–2012 survey, further supporting evidence of a tight homeostatic control of tissue levels of Mn (Aschner 1999; Aschner and Aschner 2005; Kwik-Uribe and Smith 2006). Blood Mn in our study compares with other cohorts of children residing near Mn emission sites. Children 7–11 years of age residing near a Mn mining district in Mexico had a mean blood Mn of 9.71 μg/L (95% CI: 9.16, 10.3 μg/L) (Riojas-Rodríguez et al. 2010). Similarly, children living near a ferromanganese refinery in Brazil had mean blood Mn concentration of 8.2 μg/L (range, 2.7–23.4 μg/L) (Menezes-Filho et al. 2011). These studies, however, did not report examination of a nonlinear relationship between blood Mn and cognitive function in children.

The relationship between hair Mn and child IQ in this study also demonstrated an inverse U-shaped association. Children with hair Mn concentrations > 747.0 ng/g had significantly lower IQ than children with hair concentrations between 207.2 and 747.0 ng/g (β = –3.66; 95% CI: –6.9, –0.43). Similar to the relationship we observed between blood Mn and Full Scale IQ, children with hair Mn in the lowest quartile of hair Mn (< 207.2 ng/g) had lower Full Scale IQ than children in the 2nd and 3rd quartile, but this was not statistically significant (β = –2.67; 95% CI: –5.87, 0.53). Hair typically grows 1 cm per month, thereby providing an exposure estimate of 1–6 months in our cohort. Inverse associations between hair Mn and child cognitive function have previously been reported (Menezes-Filho et al. 2011; Rodríguez-Barranco et al. 2013; Wright et al. 2006), yet nonlinear relationships were not reported. Menezes-Filho et al. (2011) found a significant inverse relationship between Full Scale IQ and children’s hair Mn (β = –5.78; 95% CI: –10.71, –0.21) after adjustment for maternal education and nutritional status. Similarly, Riojas-Rodríguez et al. (2010) found an inverse relationship between hair Mn and Full Scale IQ in school-aged children (β = –0.20; 95% CI, –0.42 to 0.02) after adjusting for hemoglobin, maternal education, age, and sex. Hair Mn concentrations have wide variability. Mean hair Mn in the Brazilian cohort was 5,830 ng/g, ranging from 100 to 86,680 ng/g (Menezes-Filho et al. 2011), whereas mean hair Mn in the Mexican cohort was 12,130 ng/g, ranging from 4,200 to 48,000 ng/g (Riojas-Rodríguez et al. 2010). Our cohort of primarily Caucasian children had geometric mean hair Mn concentrations of 416 ng/g, yet these values are comparable with those of a cohort of children 11–13 years of age in Tar Creek, Oklahoma, residing near a hazardous waste site where mean hair Mn was 471 ng/g (Wright et al. 2006). The wide variability in hair Mn concentration may be attributable to differences in exposure, pharmacokinetics, genetics, hair pigmentation, and hair sample collection and cleaning methods. Using Mn-treated hair samples, Eastman et al. (2013) found that the strength of cleaning solution decreased Mn concentration in the hair. Hair samples from the Tar Creek cohort and the CARES cohort were analyzed for Mn by the same laboratory providing a suitable cross-study comparison for hair Mn concentrations. To our knowledge, this is the first study to demonstrate evidence of a biphasic dose response for child cognition and hair Mn. The lack of a correlation between blood Mn and hair Mn was expected based on previous studies demonstrating a similar lack of correlation between the biomarkers (Menezes-Filho et al. 2011; Woolf et al. 2002). The lack of correlation may indicate the unique exposure routes to Mn and its pharmacokinetics leading to accumulation in hair and presence in blood.

Our findings are consistent with a dual role of Mn as a nutrient and neurotoxicant. Mn operates as a co-factor for metalloenzymes, such as superoxide dismutase (MnSOD), facilitating a critical role in oxidative stress protection (Martinez-Finley et al. 2013). It also plays a strong role in iron absorption and transport (Chandra and Shukla 1976; Garcia et al. 2007; Kwik-Uribe and Smith 2006). Mn deficiency has not been described in humans because it is ubiquitous in the diet; however, low levels of Mn have been associated with poor bone growth, skeletal abnormalities, ataxia, and abnormal glucose tolerance in animal studies (Keen et al. 1999). Mn overload was first described as “manganese crushers’ disease” and its documented neurotoxicity was characterized by symptoms of spasmodic laughter, mask-like face, peculiar gait, and hypertonia (Couper 1837; Rodier 1955). More recently, Mn overload has been referred to as “manganism” (Calne et al. 1994; Carolei et al. 1995). Occupational Mn exposure has been associated with early development of idiopathic Parkinson’s Disease or parkinsonism (Olanow 2004; Racette et al. 2012; Witholt et al. 2000).

Child residence in either the Marietta or Cambridge communities was not a significant factor in the regression analyses. Given the wide distribution of child residences within the Marietta community and their proximity to the ferromanganese refinery (Haynes et al. 2012), this finding is not surprising.

Blood Pb was significantly associated with Processing Speed, but not with Full Scale IQ or any of the other subscales. Our study population had blood Pb concentrations near the mean blood Pb of children in the nation 6–11 years of age in 2011–2012 (GM = 0.97; 95% CI: 0.88, 1.07) (CDC 2014). Although blood Pb concentrations have dramatically decreased in the United States due to its elimination from gasoline and lead-based paint, young children may still be exposed to lead by hand-to-mouth activity in homes with deteriorated lead-based paint (Bornschein et al. 1985; Jones et al. 2009; Manton et al. 2000). Recently it was reported that average IQ scores were 3.7 points lower in children 6 years of age with blood Pb concentrations between 5 and 9 μg/dL with children with blood lead concentrations < 5 μg/dL (Jusko et al. 2008). Lead remains a significant neurotoxicant because no threshold for Pb exposure on children’s cognitive development has been identified (CDC 2012).

Cotinine was significantly associated with Full Scale IQ. The subscales of Perceptual Reasoning, Working Memory, and Verbal Comprehension were significantly associated with serum cotinine concentrations. These findings suggest that even at very low serum cotinine concentrations, secondhand tobacco smoke can negatively impact child cognitive function. Cotinine is a primary metabolite of nicotine and a widely used biomarker for exposure to nicotine (Benowitz et al. 2009). Using the NHANES III data, Yolton et al. (2005) found a significant inverse association between ETS exposure, as measured by serum cotinine, and cognitive deficits among children (6–16 years of age), even at very low levels (GM = 0.23 ng/mL).

The volunteer selection method for enrollment in the study limits generalizability to the general population. Yet the negative associations between child IQ and our confounders, including exposure to other neurotoxicants, parenting style, education, and IQ, contributes to confidence in the associations found.

To our knowledge, this is the first study to demonstrate the inverted U-shaped association with both hair Mn and blood Mn and cognition in children. These findings add to the growing body of evidence suggesting that Mn exposure affects child intellectual development. Inclusion of multiple neurotoxicants in this study provided a robust analysis between Mn exposure and intellectual function in children because we were able to adjust for potential confounding by Pb and ETS. Future studies of Mn exposure should include other neurotoxicants, particularly cotinine and Pb, when examining the impacts of Mn exposure in pediatric populations.
